# The Feasibility of Omission of Postoperative Radiotherapy in Japanese Patients With Early Breast Cancer Treated With Breast-Conserving Surgery

**DOI:** 10.7759/cureus.60228

**Published:** 2024-05-13

**Authors:** Akihiro Nakashima, Hideya Yamazaki, Gen Suzuki, Kei Yamada, Norihiro Aibe, Takuya Kimoto, Koji Masui, Katsuhiko Nakatsukasa, Tetsuya Taguchi, Yasuto Naoi

**Affiliations:** 1 Radiology, Kyoto Prefectural University of Medicine, Kyoto, JPN; 2 Radiology, Graduate School of Medical Science, Kyoto Prefectural University of Medicine, Kyoto, JPN; 3 Endocrine and Breast Surgery, Kyoto Prefectural University of Medicine, Kyoto, JPN

**Keywords:** breast-conserving surgery, invasive carcinoma, omission of postoperative radiotherapy, postoperative radiotherapy, breast cancer

## Abstract

Background

This study was aimed at analyzing the impact of postoperative radiotherapy (PORT) after breast-conserving surgery (BCS) on Japanese patients with early-stage breast cancer and exploring the potential of PORT omission.

Materials and methods

Data from 794 patients with early-stage breast cancer (T1-2, N0-1), who underwent BCS with (n = 310) or without PORT (n = 484) were retrospectively analyzed. Local control (LC) rate and breast cancer-specific survival (BCSS) were compared between the groups that received and did not receive PORT in the whole cohort and low-risk cohort (i.e., the cohort with negative surgical margin, lymph node negativity, and estrogen receptor positivity, excluding young age of 49 or less), and in low-risk subgroup using propensity-score matching.

Results

PORT was associated with better LC but not BCSS in the total population. In the low-risk cohort, the incidence of local recurrence in patients without and with PORT was 5.3% and 4.8%, respectively, at 10 years (p = 0.591), and 7.8% and 4.8%, respectively, according to propensity-score matching (p = 0.485).

Conclusion

PORT improved LC in the total population, but not BCSS or overall survival (OS). In the low-risk group analysis (negative surgical margin, lymph node negativity, estrogen receptor positivity, and age 50 years or more), equivalent LC, BCSS, and OS were found including propensity-matched comparison. Therefore, this study showed that the omission of PORT could be a treatment option for low-risk Japanese patients. Further multi-center prospective studies are warranted to validate these findings and reduce the unnecessary burden of PORT for patients and institutions.

## Introduction

In the world, female breast cancer has become the second most often diagnosed cancer. In 2022, there were expected to be 2.3 million new cases [1]. Postoperative radiotherapy (PORT) is the established standard of care following breast-conserving surgery (BCS) for early breast cancer because it enhances local recurrence-free survival and reduces cancer-specific mortality, as supported by several randomized clinical trials (RCTs) and meta-analyses [2-4].

Some patients do not undergo PORT for various reasons, including concerns regarding secondary cancer and side effects, such as breast pain, dermatitis, risk for heart and lung complications, lack of informed consent, or fragile condition [5,6]. Therefore, omission of PORT has been explored in several low-risk groups, more specifically in those with early disease(s) (T1-2, N0-1), advanced age, and/or hormone positivity, in part, due to the limited benefits of PORT [6-13]. Recently, several retrospective studies and prospective RCTs in Western countries have indicated that omitting PORT in elderly patients is a viable option [6-13]. For example, the PRIME II trial on BCS with or without irradiation demonstrated the safety of the omission of PORT in women ≥65 years of age with T1 or T2 tumors (≤3 cm) and node-negative, estrogen receptor (ER)-positive breast cancer [6]. However, such evidence is limited in non-Asian cohorts, and Asian and non-Asian people differ in the effectiveness and toxicity of systemic therapy [14].

Therefore, we conducted a retrospective review of data housed in our database. This study analyzed the impact of PORT on Japanese patients with early breast cancer who underwent BCS to explore the potential of omitting PORT.

## Materials and methods

We conducted a retrospective analysis of 1137 Japanese patients who had BCS for early breast cancer at the Department of Endocrine and Breast Surgery, Kyoto Prefectural University of Medicine, between January 2001 and May 2016. We included histologically confirmed breast cancer patients with clinical T1-2N0-1M0 disease, identified based on available and accessible data on the T, N, and M classifications according to the National Comprehensive Cancer Network (NCCN) risk classification [15] and hormonal receptor status. We excluded patients with distant metastases at diagnosis (n = 10), Tis or 3-4 (n = 165), node-positive (N2 or more, n = 152), short follow-up within six months without any failure (n = 11), and incomplete PORT discontinuation before the planned dose (n = 5). A total of 794 patients who underwent BCS with or without PORT were eligible for analysis. Patient characteristics are shown in Table 1.

**Table 1:** Patients and treatment characteristics. * Comparison was made between RT (+) and RT (-) groups. cT: clinical T category; cN: clinical N category; ER: estrogen receptor; PgR: progesterone receptor; HER2: human epidermal growth factor receptor 2; PORT: postoperative radiotherapy; RT: radiotherapy.

Factor	Group	Total (n = 794), No. (%) or median (range)	PORT (-) (n = 484), No. (%) or median (range)	PORT (+) (n = 310), No. (%) or median (range)	p-value*
Age		53.00 (23.00, 89.00)	56.00 (26.00, 89.00)	50.00 (23.00, 82.00)	<0.001
cT	1	371 (46.7)	224 (46.3)	147 (47.4)	0.771
	2	423 (53.3)	260 (53.7)	163 (52.6)	
cN	0	705 (88.8)	421 (87.0)	284 (91.6)	0.05
	1	89 (11.2)	63 (13.0)	26 (8.4)	
ER	Positive	555 (69.9)	317 (65.5)	238 (76.8)	0.001
	Negative	239 (30.1)	167 (34.5)	72 (23.2)	
PgR	Positive	433 (62.8)	231 (57.9)	202 (69.7)	<0.001
	Negative	256 (37.2)	253 (52.3)	108 (34.8)	
HER2	Positive	84 (10.6)	50 (10.3)	34 (11.0)	0.813
	Negative	710 (89.4)	434 (89.7)	276 (89.0)	
Margin	Positive	219 (27.6)	77 (15.9)	142 (45.8)	<0.001
	Negative	575 (72.4)	407 (84.1)	168 (54.2)	
Preoperative systemic therapy	Yes	148 (18.6)	88 (18.2)	60 (19.4)	0.709
	No	646 (81.4)	396 (81.8)	250 (80.6)	
Adjuvant chemotherapy	Yes	159 (20.0)	95 (19.6)	64 (20.6)	0.785
	No	635 (80.0)	389 (80.4)	246 (79.4)	
Adjuvant hormonal therapy	Yes	331 (41.7)	184 (38.0)	147 (47.4)	0.01
	No	463 (58.3)	300 (62.0)	163 (52.6)	
Follow-up period (months)		91.0 (6.6, 197.8)	96.8 (6.6, 197.8)	88.9 (6.6, 178.8)	0.005

A total dose of 50 Gy was administered as PORT in 2 Gy fractions daily in two opposing tangential fields to the residual breast tissue utilizing generally 6-MV photons. Cases with a pathologically positive surgical margin received an additional boost radiotherapy with 10 Gy in five fractions using electron beams (4-12 MeV) in general.

In general, adjuvant endocrine therapy for early breast cancer in pre-menopausal women included tamoxifen (20 mg/day) ± goserelin (3.6 mg/four weeks) or leuprorelin (3.75 mg/four weeks), whereas in post-menopausal women, it included tamoxifen (20 mg/day), aromatase inhibitors (anastrozole 1 mg/day or letrozole 2.5 mg/day), or toremifene (40 mg/day). The standard administration period of adjuvant endocrine therapy was five years or more (up to 10 years). Preoperative systemic therapy mainly consisted of the cyclophosphamide/epirubicin/fluorouracil and taxane regimens, with or without trastuzumab.

The primary endpoint was local control (LC), and local recurrence was defined as any cancer in the scar or the same breast (including a second primary tumor in the same breast) [6]. The secondary endpoints were breast cancer-specific survival (BCSS) rate and overall survival (OS) rate. We examined factors influencing LC, BCSS, and OS in the total population. Next, we compared LC, BCSS, and OS between the groups that received PORT (RT (+)) or did not receive PORT (RT (-)) in the total population. We added subgroup analysis of the low-risk cohort and a propensity-matched group in the low-risk cohort, which consisted of surgical margin-free (>5 mm), lymph node-negative, and ER-positive cases, and age 50 or more according to previous literature [6-10].

Informed consent was obtained by providing an opt-out form on the website, and patients who refused to participate were excluded (number of people excluded = 0). We also received permission to analyze and publish data according to the guidelines of the Institutional Ethics Committee (approval number: ERB-C-1363-1). We published another article [16] using a different population of the same database with this approval number.

Statistical analyses

The EZR (Easy R) statistical package was used for statistical analyses [17]. Fisher's exact test and Student's t-test were used to analyze percentages and normally distributed data, respectively. The Mann-Whitney U test was used for comparisons of skewed data. Kaplan-Meier analysis was performed to analyze the LC, BCSS, and OS. The time to event was determined from the day of surgery. Cutoff values were set as the median or mean if they were not specified. Multivariate Cox regression models were applied for LC, BCSS, and OS. The candidate covariates in these models were age (≤ 65 vs. > 65), clinical T classification (T1 vs. T2), clinical and pathological N classification, estrogen receptor (ER) status, progesterone receptor (PgR) status, human epidermal growth factor receptor 2 (HER2) status, margin status, preoperative systemic therapy, adjuvant chemotherapy, and adjuvant hormonal therapy and PORT. Variable selection for multivariate models was conducted using the stepwise method with the Akaike information criterion. Statistical significance was set at p < 0.05.

As we did not randomize the included patients, there were unbalanced baseline characteristics that could lead to selection bias. Therefore, this selection bias influenced the decision to administer or not administer PORT. We used the propensity score, which was defined as the probability of being assigned to the RT (-) or RT (+) group, given the patient characteristics. The logistic regression model was used to calculate the propensity scores, considering the baseline covariates mentioned above excluding PORT. After the initial analysis of the whole and low-risk cohorts, we performed a propensity score-matched pair analysis (a caliper value of 0.2 was used) to minimize bias related to the choice of and allocation to the RT (-) or RT (+) groups in the low-risk cohort. Nine factors for the low-risk cohort prescribed before were used to create a 1:1 matched cohort assigned to the RT (+) and RT (-) groups.

## Results

Patient characteristics and factors influencing local control, breast cancer-specific survival, and overall survival in the total population

The median follow-up was 7.8 years (range: six months to 16.5 years). Patient characteristics and treatments are summarized in Table 1. The PORT group consisted of younger patients with more advanced disease stages and positive margins. The outcome after BCS is depicted in Table 2. PORT was associated with better LC in the total population.

**Table 2:** Outcomes after BCS. * Comparison was made between RT (+) and RT (-) groups. BCS: breast-conserving surgery; RT: radiotherapy.

Factor	Group	Total (n = 794), No. (%)	RT (-) (n = 484), No. (%)	RT (+) (n = 310), No. (%)	p-value*
Local failure	Yes	55 (6.9)	46 (9.5)	9 (2.9)	<0.001
Lymph node recurrence	Yes	23 (2.9)	13 (2.7)	10 (3.2)	0.669
Distant metastasis	Yes	30 (3.8)	19 (3.9)	11 (3.5)	0.851
Cause of death	Death of breast cancer	21 (2.6)	14 (2.9)	7 (2.3)	0.751
	Other causes of death	5 (0.6)	5 (1.0)	0 (0.0)	0.181

**Figure 1:**
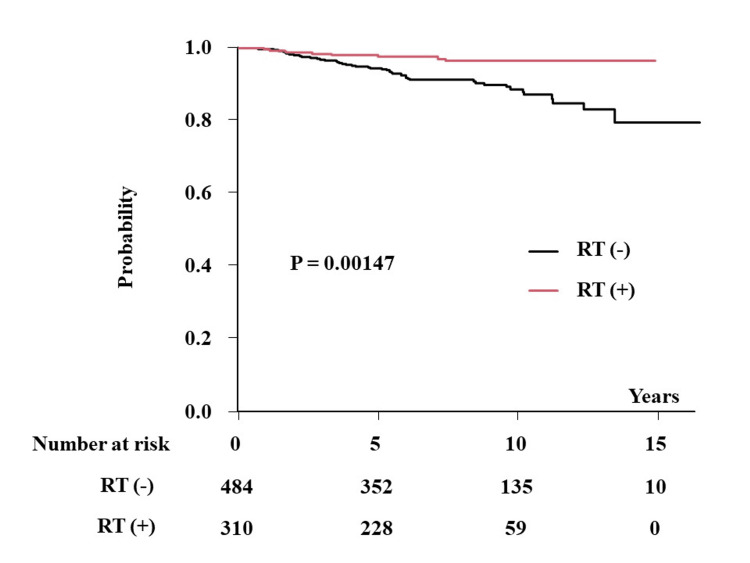
Local control after breast-conserving surgery according to the presence or absence of postoperative radiotherapy. RT: radiotherapy.

The incidence of local recurrence was 11.3% (95% CI = 8.3-15.4%) and 3.7% (95% CI = 1.9-7.3%) at 10 years (5.6% (5.8-8.2) and 2.5% (1.2-5.2%) at five years) in patients without and with PORT, respectively (p = 0.00147) (Figure 1). BCSS was 96.6% and 95.9% at 10 years (p = 0.924) (98.6% (96.9-99.4%) and 98.5% (96.1-99.4%) at five years) in patients with and without PORT, respectively. OS was 95.3% and 95.9% at 10 years (p = 0.521) (95.3% (92.0-97.3%) and 95.9% (90.5-98.3%) at five years) in patients with and without PORT, respectively. Multivariate Cox analysis revealed that ER positivity and PORT administration were statistically significant predictors of LC (Table 3). Regarding BCSS and OS, PORT administration was not a significant predictor.

**Table 3:** Multivariate analysis for LC, BCSS, and OS. * Comparison was made between RT (+) and RT (-) groups. LC: local control; BCSS: breast cancer-specific survival; OS: overall survival; 95% CI: 95% confidence interval; cT: clinical T category; ER: estrogen receptor; RT: postoperative radiotherapy.

Outcome	Variable	Strata	Hazard ratio (95% CI)	p-value*
LC	ER	Positive vs. negative	0.34 (0.16-0.73)	0.005
	RT	Yes vs. no	0.25 (0.11-0.58)	0.001
BCSS	cT	1 vs. 2	4.69 (1.05-20.8)	0.041
OS	cT	1 vs. 2	6.67 (1.52-29.2)	0.011

Impact of PORT on the low-risk cohort

A comparison of patient characteristics is shown in Table 4. The PORT group comprised younger patients with more advanced disease stages who underwent adjuvant hormonal therapy.

**Table 4:** Patients' treatment characteristics and outcomes in the low-risk group. * Comparison was made between RT (+) and RT (-) groups. cT: clinical T category; PgR: progesterone receptor; HER2: human epidermal growth factor receptor 2; RT: radiotherapy.

Factor	Group	RT (-) (n = 142), No. (%) or median (range)	RT (+) (n = 38), No. (%) or median (range)	p-value*
Age		62.00 (50.00, 87.00)	57.00 (50.00, 81.00)	0.007
cT	1	71 (50.0)	20 (52.6)	0.856
	2	71 (50.0)	18 (47.4)	
PgR	Positive	88 (63.3)	31 (81.6)	0.034
	Negative	51 (36.7)	7 (18.4)	
HER2	Positive	11 (8.0)	4 (10.5)	0.527
	Negative	131 (92.3)	34 (89.5)	
Preoperative systemic therapy	Yes	20 (14.1)	7 (18.4)	0.609
	No	122 (85.9)	31 (81.6)	
Adjuvant chemotherapy	Yes	7 (4.9)	1 (2.6)	1
	No	135 (95.1)	37 (97.4)	
Adjuvant hormonal therapy	Yes	67 (47.2)	27 (71.1)	0.01
	No	75 (52.8)	11 (28.9)	
Follow-up period (months)		91.35 (8.10, 183.30)	88.80 (14.90, 120.80)	0.481

**Figure 2:**
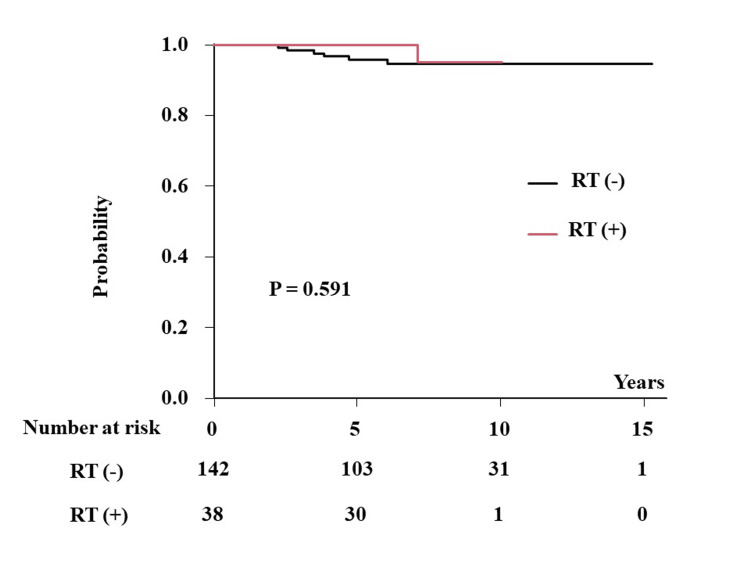
LC in the low-risk cohort according to the presence or absence of postoperative radiotherapy. LC: local control; RT: radiotherapy.

The incidence of local recurrence was 5.3% (95% CI = 2.4-11.4%) and 4.8% (95% CI = 1.5-14.7%) at 10 years (0% and 4.2% (1.8-9.8%) at five years) in patients without and with PORT, respectively (p = 0.591) (Figure 2). BCSS and OS were considered the same because there was no other cause of death; three and one deaths were due to breast cancer in patients without and with PORT, respectively. Therefore, the BCSS and OS were 96.8% and 97.4% at 10 years (100% and 99.1% (93.7-99.9%) at five years) in patients without and with PORT, respectively (p = 0.906).

Impact of PORT on the low-risk cohort according to propensity score-matched pair analysis

A propensity score-matched pair analysis was performed in the low-risk cohort. A well-matched set of 38 patient pairs in each group was generated, and the background comparison is shown in Table 5.

**Table 5:** Patients' treatment characteristics in the low-risk group after matched pair selection. * Comparison was made between RT (+) and RT (-) groups. cT: clinical T category; PgR: progesterone receptor; HER2: human epidermal growth factor receptor 2; RT: radiotherapy.

Factor	Group	RT (-) (n = 38), No. (%) or median (range)	RT (+) (n = 38), No. (%) or median (range)	p-value*
Age		55.00 (50.00, 77.00)	57.00 (50.00, 81.00)	0.399
cT	1	18 (47.4)	20 (52.6)	0.819
	2	20 (52.6)	18 (47.4)	
PgR	Positive	29 (76.3)	31 (81.6)	0.779
	Negative	9 (23.7)	7 (18.4)	
HER2	Positive	5 (13.2)	4 (10.5)	1
	Negative	33 (86.8)	34 (89.5)	
Preoperative systemic therapy	Yes	13 (34.2)	7 (18.4)	0.192
	No	25 (65.8)	31 (81.6)	
Adjuvant chemotherapy	Yes	3 (7.9)	1 (2.6)	0.615
	No	35 (92.1)	37 (97.4)	
Adjuvant hormonal therapy	Yes	26 (68.4)	27 (71.1)	1
	No	12 (31.6)	11 (28.9)	
Follow-up period (months)		88.50 (8.10, 160.00)	88.80 (14.90, 120.80)	0.685

**Figure 3:**
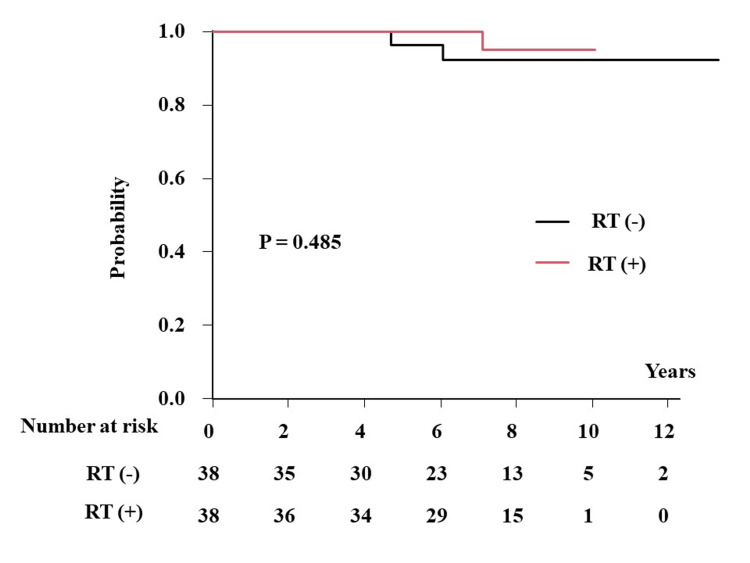
LC in matched pair population in the low-risk cohort according to the presence or absence of postoperative radiotherapy. LC: local control; RT: radiotherapy.

The incidence of local recurrence was 4.8% (95% CI = 1.7-29.3%) and 7.8% (95% CI = 2.0-27.8%) at 10 years (0% and 1.1% (0.1-6.3%) at five years) in patients with and without PORT, respectively (p = 0.485) (Figure 3). The BCSS and OS were considered the same and were 96.6% and 94.1% at 10 years (100% and 96.6% (77.9-99.5%) at five years) in patients without and with PORT, respectively (p = 0.918).

## Discussion

We examined the effect of PORT after BCS in Japanese patients with early T1-2N0-1 breast cancer to explore the impact and feasibility of omitting this therapy. We found that PORT reduced local recurrence in the total population but did not affect BCSS or OS. In the low-risk cohort, PORT did not affect all outcomes.

Our data are consistent with those of previous studies investigating LC, BCSS, and OS. For example, a meta-analysis by the Early Breast Cancer Trialists’ Collaborative Group (EBCTCG) [4] revealed a 10-year risk for local recurrence of 10.0% and 29.7% for the PORT group and controls, respectively, in women with node-negative disease and a 10-year risk for ipsilateral local recurrence of 13.1% and 46.5% for the PORT group and controls, respectively, in women with node-positive disease. According to our data, the 10-year risk for local recurrence was 9.9% and 13.6% in the PORT group and controls, respectively, among women with node-negative disease and 11.6% and 13.1% in the PORT group and the controls, respectively, among women with node-positive disease. Thus, this trend is consistent with those reported in previous investigations, although relatively better outcomes were apparent in our population [3].

Generally, guidelines including the American NCCN recommend PORT after BCS, except in patients at low risk [15]. The omission of PORT in patients with biologically low-risk tumors has been explored and established based on the presence of several low-risk biological features [6-13]; these features are generally present in elderly patients because more elderly women exhibit favorable tumor biology with low-grade, hormone receptor-positive (i.e., ER or PgR) and HER-2-negative tumors [12]. As the European Society of Breast Cancer Specialists (EUSOMA) guidelines suggest an accepted maximum rate of locoregional recurrence of 10% at 10 years for omitting PORT in elderly patients, we thought that younger patients could not be a good candidate for omitting PORT [18].

Previous studies have explored the omission of PORT. Chesney et al. conducted a systemic review and meta-analysis that included four RCTs of early-stage breast cancer treated with BCS and tamoxifen and demonstrated that PORT reduced the risk for breast and axillary recurrence in elderly women aged >70 years but did not impact distant recurrence or overall survival [11]. Kunkler et al. recently reported the highly anticipated 10-year results of the PRIME II trial and revealed that omission of PORT did not affect survival after BCS in women aged ≥65 years with T1-2 (tumors ≤ 3 cm), node-negative, and ER-positive breast cancer [6]. The authors reported a 10-year risk for local recurrence of 0.9% and 9.5% along with a 10-year OS of 80.7% and 80.8% in the PORT and control groups, respectively. Our data for the low-risk cohort exhibited local recurrence rates of 4.8% and 5.3% at 10 years and OS of 97.4% and 96.8% at 10 years in patients with and without PORT, respectively [6]. Our results regarding the benefit of omission of PORT are consistent with those of previous omission trials [6-10].

The present study had some limitations. First, as this study was not a randomized prospective study, it was difficult to address all possible biases. Our data were retrospectively obtained from a small size of the subgroups, a heterogeneous population, which may have introduced selection bias in aspects including the choice of chemotherapeutic agents. Next, histological details, such as the presence of lymph vascular invasion or the nuclear grade, were not assessed due to a lack of such information in the database. Patients with lymph vascular invasion or nuclear grade 3 tumors are not suitable as candidates for PORT omission. We speculate that in selecting suitable patients for omitting PORT, clinicians were cautious in enrolling patients with grade 3 tumors or lymphovascular invasion because the risk of local recurrence is doubled in such patients. Third, the issue of the margin status has room for discussion. Because we identified positive margins as ≤5 mm, several patients with positive margins in our cohort may have been categorized as having negative margins in other studies. Fourth, data on the prescribed dose and duration of systemic therapy (e.g., primary systemic therapy and adjuvant therapy) are lacking. At last, we could not assess the impact of the hormonal status of the patient because we did not include information on premenopausal or postmenopausal women. In the future, analyses based on genomic background intrinsic subtypes, such as luminal types, epidermal growth factor, and BRCA [5,15], which are routinely used in clinical practice, should be considered.

## Conclusions

PORT improved LC in the total population, but not BCSS or OS. In the low-risk group analysis (negative surgical margin, lymph node negativity, estrogen receptor positivity, and age 50 years or more), LC, BCSS, and OS were equivalent between RT (+) and RT (-) groups, including propensity-matched comparison. Therefore, this study showed that the omission of PORT could be a treatment option for low-risk Japanese patients. Further multi-center prospective studies are warranted to validate these findings and reduce the unnecessary burden of PORT for patients and institutions.
